# Zero-Dose Vaccination Among Children Aged 12–35 Months in Ethiopia

**DOI:** 10.1155/tswj/4373651

**Published:** 2025-11-19

**Authors:** Habtemu Jarso, Daniel Yohannes, Tsige Gebru, Lalisa Kebebe, Taye Mengistu, Demeke Tolera, Buli Teshite, Biftu Geda

**Affiliations:** ^1^Department of Public Health, Shashemene Campus of Madda Walabu University, Shashemene, Ethiopia; ^2^Department of Midwifery, Shashemene Campus of Madda Walabu University, Shashemene, Ethiopia; ^3^Family Health Unit, West Arsi Zone Health Department, Shashemene, Ethiopia; ^4^Department of Nursing, Shashemene Campus of Madda Walabu University, Shashemene, Ethiopia

**Keywords:** children, Ethiopia, 12–35 months, vaccination, zero-dose

## Abstract

**Background:**

Immunization is one of the most successful and cost-effective public health interventions worldwide. Approximately 62% of zero-dose children live in just 10 countries, including Ethiopia. In West Arsi Zone, findings from the rapid convenient survey (RCS) were inconsistent with DHIS-2 reports.

**Objective:**

The objective was to determine the proportion of zero-dose vaccination and identify associated factors among children aged 12–35 months in West Arsi Zone, June–August 2023.

**Methods:**

We conducted a community-based cross-sectional study on 1456 children selected using multistage stratified random sampling. Data were collected through face-to-face interviews with mothers/caretakers and record reviews using an ODK questionnaire. Data were cleaned and analyzed in Stata 16. Binary logistic regression identified factors associated with zero-dose vaccination. Variables with *p* < 0.25 and practical significance were included in multivariable regression; statistical significance was set at *p* < 0.05.

**Results:**

The proportion of zero-dose vaccination was 9.62% (8.10–11.13). The main reasons included unawareness of the need for vaccination (29.57%) and unavailability of the vaccines/vaccinators (15.59%). Factors associated with higher odds of zero-dose vaccination were as follows: living in kebeles where health posts lacked refrigerators (AOR = 2.08, 95% CI: 1.25–3.45), communities without social mobilization for immunization (AOR = 2.63, 95% CI: 1.44–4.80), caregiver unawareness of HEWs (AOR = 1.77, 95% CI: 1.02–3.07), poor immunization knowledge (AOR = 3.08, 95% CI: 1.83–5.21), negative (AOR = 4.24, 95% CI: 2.25–7.97), or neutral attitude (AOR = 4.21, 95% CI: 2.36–7.52) toward immunization, waiting times > 30 min (AOR = 2.83, 95% CI: 1.67–4.97), lack of health education at the facility (AOR = 2.48, 95% CI: 1.44–4.27), mothers with no ANC (AOR = 4.08, 95% CI: 2.32–7.16), home delivery (AOR = 4.07, 95% CI: 2.30–7.20), and female children (AOR = 1.75, 95% CI: 1.10–2.78).

**Conclusions:**

Zero-dose vaccination is consistent with RCS findings and is unacceptably high compared to the national targets (100% Pentavalent 1 coverage nationally; 98% per district). Interventions should focus on increasing community awareness, ensuring vaccines and vaccinator availability, and promoting equal care for male and female children.

## 1. Introduction

Vaccine-preventable diseases (VPDs) remain a major cause of child morbidity and mortality [1]. Immunization induces protective antibodies against specific diseases through vaccination [2]. It is one of the most successful and cost-effective public health interventions worldwide, contributing to the elimination or eradication of severe childhood diseases [1, 3–6].

The World Health Organization (WHO) launched the expanded program on immunization (EPI) in 1974 [7, 4, 5, 8]. Ethiopia adopted EPI in 1980 [5, 8]. Globally, childhood vaccination prevents 4 million deaths each year. Between 2021 and 2030, immunization could prevent over 50 million deaths, with measles and hepatitis B vaccines alone expected to save 19 million and 14 million lives, respectively, by 2030 [9].

Despite the achievements, more than 1.5 million people worldwide still die from VPDs each year [10]. In 2021, 18 million children remained unvaccinated (zero-dose), the highest number recorded since 2005 [9, 11]. Nearly all zero-dose vaccinated children live in low- and middle-income countries (LMICs), particularly in Africa and Southeast Asia. About 62% (11 million) of these children live in just 10 countries, including Ethiopia [1, 9, 12], which is the fourth largest contributor to the global total [11].

The Ethiopian government has demonstrated a strong commitment to expanding EPI access by training health extension workers (HEWs) and establishing 15,000 health posts [5]. However, despite promising improvements in vaccination coverage, Ethiopia still has a significant number of unvaccinated children. Between 2019 and 2021, 1.1 million children in Ethiopia received no vaccinations (zero-dose) [13]. The 2019 Ethiopian Demographic and Health Survey (EDHS) reported that 19% of children had not received any vaccines [14]. Nearly 50% of these children live in the Oromia region [11, 15].

Studies have linked zero-dose vaccination to several factors, including place of residence, place of delivery, antenatal care (ANC) follow-up, and wealth index [16, 17]. Other influencing factors include maternal age, education, employment status, parity [17], fear of COVID-19 infection at health facilities, distance, male partner involvement, and knowledge [16].

In West Arsi zone, a rapid convenient survey (RCS) revealed a significant number of zero-dose vaccinated children, which contradicts the District Health Information Software-2 (DHIS-2) data and highlights potential discrepancies. Therefore, this study is aimed at determining the prevalence of zero-dose vaccination and identifying associated factors. Unlike most previous studies in Ethiopia [18–21], which defined zero-dose as not receiving the first dose of Penta/DPT vaccine, this study defined zero-dose as the absence of any vaccine to avoid misclassification and overestimation of zero-dose prevalence [22]. Additionally, the 12–23 months age band used by previous studies [19, 20, 22, 23] overlooks children who remain unvaccinated (persistent zero-dose) even after catch-up efforts or misclassifies delayed vaccinations during the catching-up period as zero-dose. The current study addressed this issue by including children aged 12–35 months.

## 2. Conceptual Framework

**Figure 1:**
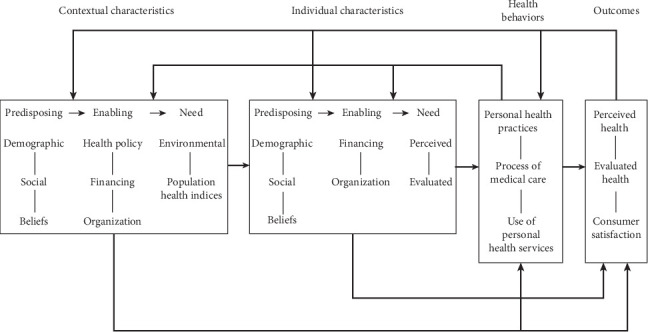
A behavioral model of health service use: incorporating contextual and individual characteristics [24].

**Figure 2:**
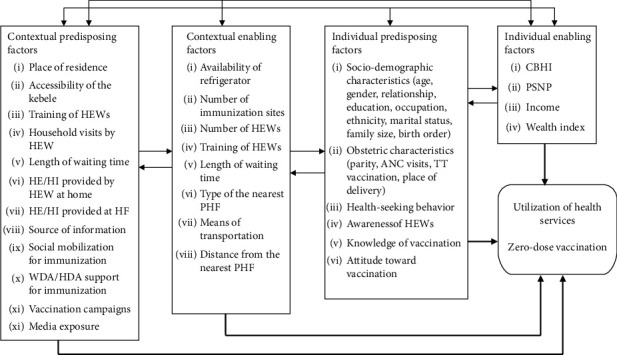
Conceptual framework of factors influencing childhood immunization utilization of West Arsi Zone (June 2023) (*Source:* adapted from ABMHSU and relevant literature).

We used Andersen's behavioral model of health service use (ABMHSU) (Figure 1) to guide the selection of explanatory variables and to develop the conceptual framework for our study (Figure 2). First developed in the 1960s, the framework has undergone four phases, with the most recent update in the 1990s. This model helps identify factors that facilitate or hinder service utilization. It explains that an individual's access to and use of health services depends on three core factors: predisposing, enabling, and need factors. Additionally, the model considers how environmental factors (such as the external environment and health system) and personal characteristics interact to influence health behavior, which in turn affects health outcomes including perceived and evaluated health status [24–34].

1. Predisposing factors

Predisposing factors refer to sociocultural characteristics that exist before an individual becomes ill [24, 25].

• Demographic characteristics: age and gender
• Social structure: education, occupation, ethnicity, social networks, social interactions, and culture
• Health beliefs: attitudes, values, and knowledge about the health care system
  2. Enabling factors

Enabling factors refer to the logistical aspects of obtaining care [24, 25].

• Personal/family: knowledge and resources to access healthcare services, income, health insurance, a regular source of care, travel, and the extent and quality of social relationships
• Community: availability of healthcare personnel and facilities, as well as waiting time
• Possible additions: genetic factors and psychological characteristics
  3. Need factors

Need factors represent the most immediate reasons for using healthcare services, arising from functional and health problems that create a demand for care [24, 25].

## 3. Methods and Materials

### 3.1. Study Setting and Design

A community-based cross-sectional study was conducted from June 23 to August 17, 2023 in the West Arsi Zone of the Oromia region. Shashemene City, located 250 km southeast of Finfinne, serves as the administrative center of the zone. The West Arsi Zone covers an area of 11,776.72 km^2^ and is divided into 13 districts (*woredas*) including Adaba, Negele Arsi, Dodola, Gedeb Hassasa, Kofele, Kokosa, Qore, Nannawa Shashemene, Nensebo, Seraro, Shala, Heban Arsi, and Wondo. Additionally, it had four administrative towns: Dodola, Kofale, Negele Arsi, and Shashemene. The population of the zone was officially estimated at 2,929,894 in mid-2022.

There were seven public hospitals, 85 health centers, and 343 health posts that routinely provide vaccination to children under five in the district. Additionally, there were also five private hospitals and several private clinics although they do not offer vaccination services.

### 3.2. Study Population

The study population consisted of 12–35 months aged children and their mothers or caretakers. This age group was selected to identify children who remain unvaccinated (persistent zero-dose) beyond the catch-up period and to avoid misclassifying delayed vaccinations during this period as zero-dose. A similar age group was also used in some previous studies [18, 21, 35], facilitating comparison of results.

Inclusion Criteria

• Residents of West Arsi Zone for at least 6 months

Exclusion Criteria

• Mothers/caretakers who were too ill to respond
• Individuals living on the street

### 3.3. Sample Size Determination

#### 3.3.1. Sample Size for Zero-Dose Vaccination

The sample size was determined using the single population proportion formula. We assumed a zero-dose vaccination prevalence of 28% in Oromia [36], a 95% confidence level, and a 4% margin of error due to a limited budget for data collection. We aimed to determine zero-dose vaccination separately for children aged 12–23 and 24–35 months, resulting in two strata. Additionally, we accounted for a maximum nonresponse rate of 10% and a design effect of 1.5 similar to previous studies [23, 37–41] due to limited resources. Based on these assumptions, the final calculated sample size was 1599.

#### 3.3.2. Sample Size for Factors Associated With Zero-Dose Vaccination

We determined the sample size for factors associated with zero-dose vaccination using the double population proportion formula. The calculation considered a 95% confidence level, 80% power, an equal ratio of unexposed to exposed, 10% nonresponse, and a 1.5 design effect. The largest sample size was 924 (Table 1).

The largest calculated sample size was 1599, which was selected for the current study.

### 3.4. Sampling Technique and Procedure

A stratified multistage random sampling technique was used. First, kebeles (the lowest administrative unit) were categorized as accessible or hard-to-reach. Then, 30% of kebeles were randomly selected from each stratum.

**Figure 3:**
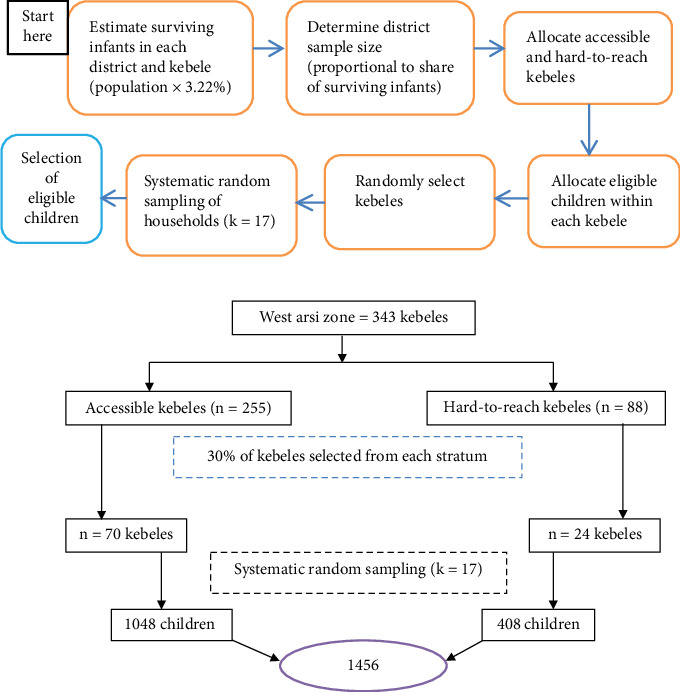
Schematic representation of sampling procedure for the study on zero-dose vaccination among children aged 12–35 months in West Arsi Zone, June 2023.

To identify eligible children, we first estimated the number of surviving infants in each district and kebele by multiplying the population by a conversion factor of 3.22%. The district-level sample size was then calculated using proportional allocation, based on each district's share of surviving infants relative to the total across all districts. The number of kebeles selected per district was determined proportionally to the district sample and further allocated between accessible and hard-to-reach kebeles according to their relative numbers in each district. Kebele selection was performed randomly using a computer-generated random number. Within each selected kebele, the number of eligible children was proportionally allocated. Households were selected using systematic random sampling, with the sampling interval (*k* = 17) calculated by dividing the total number of eligible children in the kebele by the allocated sample (Figure 3).

To ensure equal representation of children aged 12–23 and 24–35 months, selection alternated between these age groups. If a household had multiple eligible children (e.g., twins and siblings in different age groups), one child was randomly selected using the lottery method to avoid dependency in observations. Public facilities such as health centers, mosques, churches, health posts, or governmental offices served as the reference points for selecting the first household.

### 3.5. Variables

The outcome variable was zero-dose vaccination status. Explanatory variables were categorized as follows: household characteristics (family size, number of children aged 1–3 years, wealth index, residence, kebele accessibility, transportation to the nearest public health facility, productive safety net program PSNP)participation, and community-based health insurance (CBHI) membership; respondent/child characteristics (relationship to child, age, religion, ethnicity, education, marital status and usual occupation of mother/caretaker, sex of caretaker, education of father, child's age, sex, and birth order); obstetric characteristics (ANC follow-up, tetanus toxoid (TT) vaccination during pregnancy, and place of delivery); health information and health seeking behavior (sources of vaccination information, health seeking practices, receipt of health education, mother's/caretaker's awareness of HEWs, immunization knowledge, and attitudes); and service- and provider-related factors (type of nearest health facility, refrigerator availability at health posts, number of outreach immunization sites and HEWs, social mobilization activities, Women Development Army (WDA) support, and waiting time at the usual facility).

### 3.6. Data Collection Methods and Tools

Data were collected by face-to-face interviews with mothers or immediate caretakers using a semistructured questionnaire. It consisted mainly of closed-ended questions, with a few open-ended items for variables such as the age of the mother/caretaker, the child's age, and reasons for not vaccinating a child. The questionnaire was developed based on a review of the EDHS data collection tool and previous similar studies. To facilitate data collection, the questionnaire was converted into an Open Data Kit (ODK) template, downloaded onto mobile or tablet devices, and used for digital data entry. The geographic location of each household was recorded using a Global Positioning System (GPS).

Zero-dose vaccination status was assessed through mother or caretaker recall. Immunization knowledge of the mother/caretaker was measured using five questions. Each correct answer was awarded a score of 1, while incorrect or do not know answers were given a score of 0, yielding a total score of 14 points. Given that the knowledge scores were normally distributed, the mean score was used to classify participants' knowledge as good or poor following the approach of several prior studies in Ethiopia [42–46]. Attitude toward vaccination was assessed using four items on a five-point Likert scale, yielding a total score ranging from 4 to 20. Attitude was classified as positive, neutral, or negative based on the modified Bloom's cut-off points for attitude classification, as applied in previous studies [47–52].

### 3.7. Operational Definitions

• Zero-dose vaccination (unvaccinated): A child who has not received any vaccination.
  o. Knowledge of vaccination: Scores equal to or greater than the mean (≥ 5.70) were classified as indicating good knowledge, while scores below the mean (< 5.70) were classified as poor knowledge.
  o. Attitude toward vaccination: Scores equal to or greater 16 (≥80%) were classified as a positive attitude, scores between 12 and 15 (60%–79%) as neutral and scores below 12 (< 60%) as a negative attitude.

### 3.8. Data Processing and Analysis

The collected data were cleaned and analyzed using Stata 16. Descriptive statistics were computed to summarize the data. To identify the factors associated with a child's immunization status, a binary logistic regression model was fitted. Initially, univariate models were applied to each explanatory variable. Variables with a *p* value less than 0.25, as well as those with clinical or practical significance, were selected for inclusion in the multivariable regression model. Multicollinearity among independent variables was assessed using the variance inflation factor (VIF) in linear regression, and no significant multicollinearity was detected. The final multivariable model was fitted, with statistical significance declared at a *p* value less than 0.05. Model fit was assessed using the Hosmer–Lemeshow test which yielded a *p* value of 0.31, indicating good model fit. Additionally, the classification table showed an accuracy of 93.41%.

### 3.9. Data Quality Management

Data were collected by Level IV and above nursing graduates, a Bachelor of Science (BSc) graduate supervisor overseeing the process. A research team was responsible for checking the completeness and consistency of the collected data daily. To ensure quality and consistency, 2 days of training were provided to data collectors and supervisors on the sampling approach, data collection methods and tools, and ethical considerations.

To improve data quality, ODK was used. The name of the data collector and GPS coordinates were included in the template to ensure that the household was visited and the respondent was properly interviewed. The ODK template was created in both English and Afan Oromo. The questionnaire was translated into Afan Oromo by a language expert and was back-translated into English by a different language expert to ensure consistency. The Afan Oromo version of the ODK template was used for interviews. Data collectors were instructed not to have any contact with HEWs. The questionnaire was pretested, and modifications were made based on the pretest results before the actual data collection began.

### 3.10. Ethical Considerations

Ethical approval for the study was obtained from the Research Ethics Committee of Shashemene Campus, Madda Walabu University. A support letter was also secured from West Arsi Zone Health Department and the respective District Health Offices. The study adhered to the fundamental ethical principles of respect for persons, beneficence/nonmaleficence, and justice. Informed verbal consent was obtained from all respondents. Confidentiality of the information gathered was strictly maintained, and no personal identifiers, such as names or other identifying data, were recorded. Participation in the study was completely voluntary, with participants being informed of their right to refuse participation or to discontinue the interview at any time.

## 4. Results

### 4.1. Kebele/Health Post Characteristics

**Figure 4:**
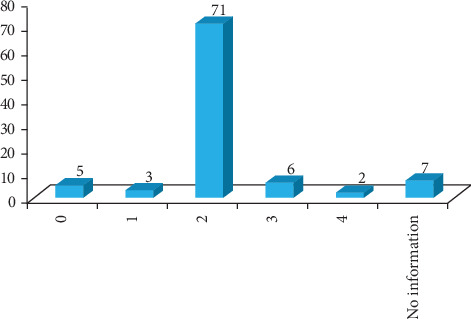
Distribution of number of outreach EPI service sites per health post, the West Arsi Zone, June 23 to August 17, 2023.

**Figure 5:**
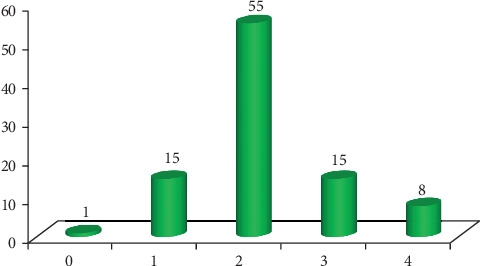
Distribution of number of health extension workers per health post, the West Arsi Zone, June 23 to August 17, 2023.

A total of 94 kebeles were surveyed, including 70 accessible and 24 hard-to-reach kebeles. Half (47) of the kebeles' health posts lacked a refrigerator. Most health posts (71) had two outreach EPI service sites (Figure 4). Most health posts (55) had two HEWs, while one had none (Figure 5). All HEWs had received integrated refresher training (IRT) within the last 2 years.

### 4.2. Household, Respondent, and Child Characteristics

A total of 1456 respondents participated in the survey, resulting in a 91.06% response rate. Among them, 1048 were from accessible kebeles, while 408 were from hard-to-reach kebeles. The majority (74.9%) lived in the rural areas and most (93.4%) were females. More than half (53.6%) were aged 25–34 years, with a mean age of 29.8 (SD = 7.4). Nearly all respondents (94.6%) identified as Oromo and most (82.1%) were Muslim. Nine in 10 respondents (90%) were mothers. Most respondents were married (96.7%) and nearly three-quarters (74.0%) were housewives. A little over one-fifth (21.7%) of children's mothers/caretakers had completed Grades 5–8, while 29.74% of the children's fathers had attended the same grade levels. More than half of the respondents (52%) lived in households with more than five family members, with the average family size of six. In 81.5% of households, there was only one child aged 1–3 years. Most households (78%) were enrolled in the CBHI program. Children aged 12–23 months accounted for 51.9% of the participants in the study, while males represented 56.3% of the enrolled children. Most of the enrolled children (81.5%) were second-born or higher in birth order (Table 2).

### 4.3. Obstetric, Health Information, and Health-Seeking Characteristics

Most mothers of the enrolled children (62.2%) had given birth to 2–5 children. Additionally, 90.7% attended ANC, and 86.1% received vaccinations during the pregnancy of the index child. However, only 60% of enrolled children were born in a health facility (Table 3).

### 4.4. Zero-Dose Vaccination Status and Reported Reasons

**Figure 6:**
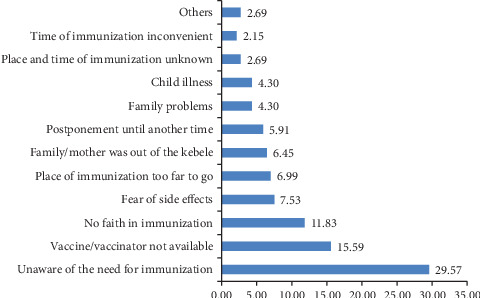
Reasons for not vaccinating their child (zero-dose), the West Arsi Zone, 23 June–17August 2023.

Nearly one in 10 children (9.6%) did not receive any vaccine doses during routine immunization, with 9.14% of children aged 12–23 months and 10.13% of children aged 24–35 months remaining unvaccinated. The main reported reasons for zero-dose vaccination among children included a lack of awareness about the need for vaccination (29.57%), unavailability of vaccines or vaccinators (15.59%), distrust in vaccination (11.83%), fear of side effects (7.53%), and long distance to the vaccination site (6.99%) (Figure 6).

**NB**:

• Family problems include illness of the care taker and mother too busy.
• Other reasons include being corona time, all births at home, never taken child to health facility, old age, and being street resident.

### 4.5. Factors Associated With Zero-Dose Vaccination

Factors either significantly associated with zero-dose vaccination (*p* value < 0.05) in bivariate binary logistic regression or candidates for multiple binary logistic regression (*p* value < 0.25) were presented in the following table (Table 4).

However, the type of health facility nearest to the respondent's residence, house ownership, respondent's relationship to the index child, respondent's gender, ethnicity, family size, number of live births, enrollment in the PSNP, age of the index child, and birth order of the index child were not significantly associated with zero-dose vaccination.

A total of 24 variables were selected based on the *p* value of less than 0.25 and their public health importance and were included in multivariable binary logistic regression. This was because there were 140 children who were zero-dose vaccinated and 1316 children who were partially or fully vaccinated. The following factors were found to be statistically significantly associated with zero-dose vaccination: availability of a refrigerator at the health post, presence of social mobilization for immunization, age of respondent, sex of the index child, ANC during the pregnancy of the index child, place of birth of the index child, waiting time at the health facility, respondent knowledge of the HEW, respondent's knowledge about immunization, respondent's attitude towards immunization, respondent receiving health education at the health facility, enrollment in the CBHI, and the wealth index (Table 5).

### 4.6. Reasons Reported by HEWs for Zero-Dose Vaccination

HEWs who attended IRT on June 14, 2023 were asked to report reasons for zero-dose vaccination. A total of 135 responses were obtained from over 50 HEWs across the Shashemene, Shalla, Siraro, and Wondo districts.

The leading reported reason for zero-dose vaccination was the migration of residents for various reasons, including moving to the city in search of work due to the famine, migrating to the lake in search of water, and fleeing the area due to security issues. The second most common reason cited was the work overload of HEWs and inadequate human resources. HEWs mentioned that there was often no HEW in each kebele zone, and having only one or two HEWs was insufficient to cover a kebele with more than 10,000 people.

The third most common reason was health worker-related issues such as failure to communicate the benefits of immunization correctly, failure to vaccinate on a daily basis (health post not being open on some days), not utilizing outreach sites, lack of attention to vaccination, postponing *Bacillus* Calmette–Guerin (BCG) vaccinations to other days, lack of follow-up by health centers, HEWs being absent on scheduled appointment dates, and miscommunication of vaccine side effects.

The fourth leading reason is lack of transportation and distance. The fifth reason involved awareness and attitude-related issues, including uneven community perceptions, the mothers refusing to bring children for vaccination by claiming that “the child cries or gets fever after vaccination” or “the child has an evil eye.” A few mothers or caretakers even consider immunization unnecessary because it is free.

Other reported reasons included a lack of logistics, such as unavailable vaccines or refrigerators. Additionally, maternal or caretaker factors like a mother becoming ill after delivery, forgetting appointments, not attending outreach sessions, or mothers or caretakers taking children to the market were also reported by HEWs.

## 5. Discussion

We conducted a community-based cross-sectional study from June 23 to August 17, 2023, in the West Arsi Zone, Oromia region, to determine the prevalence of zero-dose vaccination and identify associated factors among children aged 12–35 months. We found that one in 10 children had not received any vaccinations. The main reasons reported by respondents for zero-dose vaccination were a lack of awareness about the need for vaccination and the unavailability of vaccines or vaccinators.

Factors independently associated with zero-dose vaccination included the availability of a refrigerator at the health post, the presence of social mobilization for immunization, age of the respondent, sex of the index child, ANC during pregnancy, the index child's place of birth, waiting time at the health facility, whether the respondent knew the HEWs, the respondent's knowledge about immunization, the respondent's attitude towards immunization, whether the respondent received health education at a health facility, enrollment in the CBHI, and wealth index.

The level of zero-dose vaccination in the current study (9.1% among children aged 12–23 months and 9.6% among children aged 12–35 months) was substantially lower than previous estimates. For example, a study using 2019 Mini EDHS data for children aged 12–35 months reported 46.5% DPT/Penta zero-dose [21], and a national study conducted among pastoralist, hard-to-reach, conflict-affected, urban slum, and special populations (internally displaced populations and refugees) reported 33.7% DPT/Penta zero-dose among children aged 12–35 months [11, 18]. Our findings were also lower than the 24.8% national (27.8% in Oromia) DPT/Penta zero-dose reported by a study using Ministry of Health data for children aged 12–23 months [19] and the 23.7% DPT/Penta zero-dose reported by using EDHS data for the same age group [20]. Likewise, our results were lower than the 27.8% national (28% in Oromia) “truly zero-dose” reported by a study using EDHS data of children aged 12–24 months [36], 19% “truly zero-dose” reported by the 2019 Mini EDHS children among children aged 12–23 months [14], the 16.3% “truly zero-dose” children reported by a study using EDHS data of children aged 12–35 months, and the 14% “truly zero-dose” reported among pastoral and agrarian children aged 12–23 months [23].

However, the prevalence in our study was comparable to 9% “truly zero-dose” reported among agrarian children aged 12–23 months [23] and slightly higher than the 7.5% “truly zero-dose” reported in a multicountry analysis of 82 LMICs [22].

These differences are expected, as variations in time and interventions to improve immunization coverage can influence vaccination prevalence. Differences in the definition of zero-dose, age groups studied and study settings may also contribute to the variations. Despite improvements in childhood immunization coverage in the zone, the level of zero-dose vaccination is consistent with RCS findings, and is much higher than the targets set in the national EPI Comprehensive Multi-Year Plan, which aims to achieve 100% Pentavalent 1 coverage nationally and 98% in every district [53].

Regarding factors associated with zero-dose vaccination, the odds of zero-dose vaccination were twice as high for a child from a kebele without a refrigerator at the health post compared to a child from a kebele with a refrigerator. This may be because vaccines and vaccinators are more likely to be available at health posts with refrigerators. Additionally, respondents identified the lack of vaccines or vaccinators as one of the main reasons for zero-dose vaccination, which is consistent with a previous study [23].

Similarly, the odds of zero-dose vaccination were two times higher for a child whose respondent reported waiting more than 30 min at a health facility compared to a child whose respondent reported waiting 30 min or less. This may also be due to a lack of vaccines or vaccinators at the health facility. Previous studies have also reported that caregivers were discouraged from vaccinating their children by long waiting times resulting from the open-vial policy, which requires a certain number of children to be present before administering the BCG vaccine [54]. The odds of zero-dose vaccination were three times higher for a child from a kebele without social mobilization for immunization than for a child from a kebele with social mobilization for immunization. Similarly, the odds were 1.4 times higher for a child whose respondent did not know HEWs compared to a child whose respondent knew HEWs. Previous studies also reported that children from households not visited by HEWs had significantly higher odds of zero-dose [23].

Additionally, the odds of zero-dose vaccination were two times higher for a child whose respondent did not receive health education at a health facility than for a child whose respondent received it. A child whose respondent had poor knowledge about immunization had three times higher odds of zero-dose vaccination than a child whose respondent had good knowledge. Likewise, the odds were six times higher for a child whose respondent had a negative attitude toward immunization and four times higher for a child whose respondent had a neutral attitude, compared to a child whose respondent had a positive attitude, which is consistent with a previous study [19].

The odds of zero-dose vaccination were also 2.5 times higher for a child whose mother did not attend ANC during pregnancy than for a child whose mother did. This finding is consistent with previous studies, which reported that lack of ANC or lower frequency of ANC visits significantly increases the odds of zero-dose vaccination [18–22, 35, 36, 55]. Similarly, children born at home had four times higher odds of zero-dose vaccination than those born in a health facility as reported in the previous studies [20, 22, 23, 35, 36, 55]. These findings indicate that inadequate information and poor awareness about immunization among parents/caretakers increase the likelihood of zero-dose vaccination among children.

The odds of zero-dose immunization were twice as high for a child from a household not enrolled in a CBHI scheme compared to a child from a household that was enrolled. This may be because parents or caretakers associated vaccination with payment, even though it was free, suggesting a lack of information about immunization among respondents. The odds of zero-dose vaccination were 3.8, 2.7, and 4.0 times higher for children from households in the poorest, middle-lower, and middle-upper wealth index categories, respectively, compared to children from households in the richest wealth index category. This may be because households in the low economic categories do not prioritize immunization. These findings are consistent with previous studies reporting that populations with lower socioeconomic status (SES) have high numbers of children who received no vaccinations [22, 23, 35, 56].

Additionally, female children were almost twice as likely as male children to have received zero doses of vaccines, which is consistent with a previous study [20]; possibly because the community pays less attention to female children. Children whose respondents aged 15–24 years were also twice as likely as children whose respondents aged 25–34 years to have zero-dose vaccination, which is consistent with a previous study [18]. This could be because adolescent and young adult caregivers often have less experience in childcare, low levels of awareness and empowerment, and face greater socioeconomic barriers.

Although not observed in our study, previous studies have reported that rural children [20–22, 36], children from uneducated mothers/caregivers [35, 36] and children from low community level media exposure [35], had higher odds of zero-dose vaccination.

The strength of this study is that it was community-based and included both hard-to-reach and accessible kebeles in a proportionally representative way. However, the study is not without limitations. Because of budget constraints for data collection, we used a 4% margin of error and a design effect of 1.5 which may have affected the adequacy of the sample size. Furthermore, although we initially planned to survey 104 kebeles (27 hard-to-reach and 77 accessible) from 13 woredas and three town administrations, we were able to survey only 94 kebeles (24 hard-to-reach and 70 accessible) from 12 woredas and three town administrations. These may have affected the true picture of children's vaccination status. Additionally, reliance on recall may have introduced misclassification bias (overestimation of vaccinated children) due to recall or social desirability biases.

## 6. Conclusions

Although the use of recall (history) may have overestimated vaccination status, the level of zero-dose vaccination confirmed the findings of RCSs conducted by the zonal health department. This finding is unacceptably high compared with targets set in the national EPI Comprehensive Multi-Year Plan, which aims to achieve 100% Pentavalent 1 coverage nationally and 98% in every district.

Regarding the factors associated with the vaccination status of the children, reasons reported by respondents in this study, by HEWs during training conducted 2 weeks prior to data collection for the current study and by logistic regression analysis were generally consistent. The main reasons for zero-dose vaccination included unawareness of the need for vaccination, unawareness of the need for the next dose, and the absence of vaccines or vaccinators. Factors associated with vaccination status were primarily related to the availability and accessibility of vaccines and vaccinators, exposure to information about immunization, and household wealth status. Less attention paid to female children by the community and caregiver's young age were also important factors associated with zero-dose vaccination.

## 7. Recommendations

To improve the immunization status of children in the zone, the following should be done:

1. Ensure the availability of vaccinators (health workers) and an adequate supply of vaccines at health facilities nearby health facilities.
2. Strengthen community mobilization and awareness creation about the EPI.
3. Provide clear, written instructions to caregivers regarding when and where to vaccinate the children.
4. Promote gender equity in childcare emphasizing that male and female children deserve equal attention and care.
5. Raise awareness, empower adolescent and young adult caregivers, and reduce socio-economic barriers to childhood immunization.

## Figures and Tables

**Table 1:** Sample size for the factors associated with zero-dose vaccination in West Arsi zone, June 2023.

**Factors**		**Proportion among unexposed**	**Proportion among exposed**	**Sample size**	**References**
Place of residence (urban vs. rural)		10.7	33.65	198	[36]
ANC visit (yes vs. no)		18.6	54.3	111	
Place of delivery (home vs. health facility)		19.9	51.0	144	
Family planning (yes vs. no)		12.2	36.7	185	
Wealth index (rich vs. poor)		12.5	40.5	150	
Parity (0–3 vs. ≥ 6)		23.9	35.1	924	
Educational level	(no education vs. primary)	23.4	36.6	678	
	(no education vs. secondary+)	9.6	36.6	150	

**Table 2:** Distribution of respondent or child characteristics, the West Arsi Zone, June 23–August 17, 2023.

**Variable**	**Category**	**Number**	**Percent**
Age	15–24	287	19.7
	25–34	780	53.6
	35–44	326	22.4
	45+	63	4.3

Ethnicity	Oromo	1377	94.6
	Sidama	39	2.7
	Amhara	18	1.2
	Others	22	1.5

Religion	Muslim	1195	82.1
	Protestant	146	10.0
	Orthodox	108	7.4
	Catholic	7	0.5

Current marital status	Married	1408	96.7
	Widowed	20	1.4
	Divorced	18	1.2
	Separated	10	0.7

Occupation	House wife	1077	74.0
	Farmer	161	11.1
	Merchant	83	5.7
	Gov't employee	69	4.7
	Daily laborer	52	3.6
	Student	10	0.7
	Other	4	0.3

Family size	1–3	194	13.3
	4–5	505	34.7
	> 5	757	52.0

**Table 3:** Obstetric characteristics and health service utilization of the respondents, the West Arsi Zone, 23 June–17August 2023.

**Variable**	**Category**	**Number**	**Percent**
Number of live birth by the mother	1	196	13.5
	2–5	905	62.2
	> 5	355	24.4

Number of ANC attended by the mother	1	89	6.7
	2–3	795	60.2
	≥ 4	437	33.1

Number of vaccination during the pregnancy	1	307	24.5
	2	506	40.4
	3	440	35.1

**Table 4:** Factors either significantly associated with zero-dose vaccination in bivariate binary logistic regression or candidates for multiple binary logistic regression in children aged 12–35 months, the West Arsi Zone, 23 June–17 August 2023.

**Variables**		**Vaccination status**				**COR (95% CI)**	**p** ** value**
		**Zero-dose**		**Partial or full**			
		**No**	**%**	**No**	**%**		
Place of residence	Rural	97	8.9	993	91.1	1	0.111⁣^∗^
	Urban	43	11.7	323	88.3	1.36 (0.93, 1.99)	
Accessibility of the kebele	Accessible	107	10.2	941	89.8	1.29 (0.86, 1.94)	0.218⁣^∗^
	Hard-to-reach (HTR)	33	8.1	375	91.9	1	
Availability of refrigerator at health post	Yes	55	8.1	628	91.9	1	0.058⁣^∗^
	No	85	11.0	688	89.0	1.41 (0.99, 2.01)	
Number of outreach sites (*n* = 1290)	≤ 2	125	11.1	1055	88.4	6.40 (1.56, 26.23)	0.010
	3–4	2	1.8	108	98.2	1	
Number of health extension workers	1–2	27	13.9	167	86.1	1.64 (1.05, 2.58)	0.030⁣^∗^
	≥ 2	113	9.0	1149	91.0	1	
WDA/HDA supporting immunization activities	Yes	38	5.9	603	94.1	1	0.000⁣^∗^
	No	102	12.5	713	87.5	2.27 (1.54, 3.35)	
Presence of social mobilization for immun.	Yes	37	4.1	860	95.9	1	0.000⁣^∗^
	No/do not remember	103	18.4	456	81.6	5.25 (3.55, 7.77)	
Age of respondent	15–24	35	12.2	252	87.8	1.67 (1.07, 2.59)	0.023⁣^∗^
	25–34	60	7.7	720	92.3	1	
	35–44	33	10.1	293	89.9	1.35 (0.87, 2.11)	0.186
	≥ 45	12	19.0	51	81.0	2.82 (1.43, 5.58)	0.003
Sex of index child	Male	65	7.9	754	92.1	1	0.014⁣^∗^
	Female	75	11.8	562	88.2	1.55 (1.09, 2.20)	
Religion	Muslim	122	10.2	1073	89.8	1.53(0.92, 2.57)	0.102⁣^∗^
	Others	18	6.9	243	93.1	1	
Respondent's education	No formal education	82	18.6	359	81.4	3.77 (2.64, 5.39)	0.000⁣^∗^
	Has formal education	58	5.7	957	94.3	1	
Father's education	No formal education	59	18.5	260	81.5	2.96 (2.06, 4.25)	0.000⁣^∗^
	Has formal education	81	7.1	1056	92.9	1	
Current marital status	Married	128	9.1	1280	90.9	1	0.001⁣^∗^
	Others	12	25.0	36	75.0	3.33 (1.69, 6.57)	
Occupation	Farmer	11	6.8	150	93.2	2.46 (0.53, 11.39)	0.251
	Housewife	111	10.3	966	89.7	3.85 (0.93, 15.93)	0.063
	Employee	2	2.9	67	97.1	1	
	Merchant	6	7.2	77	92.8	2.61 (0.51, 13.37)	0.250
	Others	10	15.2	56	84.8	5.98 (1.26, 28.44)	0.025
Wealth index (calculated from 21 asset variables)	Poorest	36	11.3	284	88.8	2.51 (1.32, 4.75)	0.005⁣^∗^
	Middle lower	27	10.4	233	89.6	2.29 (1.17, 4.47)	0.015
	Middle	21	7.1	273	92.9	1.52 (0.76, 3.05)	0.237
	Middle upper	42	14.4	249	85.6	3.34 (1.78, 6.26)	0.000
	Richest	14	4.8	277	95.2	1	
ANC during pregnancy of index child?	Yes	74	5.6	1247	94.4	1	0.000⁣^∗^
	No	66	48.9	69	51.1	16.12 (10.69, 24.31)	
TT vaccination during ANC follow-up	Yes	66	5.3	1187	94.7	1	0.000⁣^∗^
	No	74	36.5	129	63.5	10.32 (7.07, 15.06)	
Index child's place of birth	Health facility	26	3.0	850	97.0	1	0.000⁣^∗^
	Home	114	19.7	466	80.3	8.0 (5.15, 12.43)	
Taken index child to the health facility for medical reason when the child was ill	Yes	73	7.1	955	92.9	1	0.000⁣^∗^
	No	67	15.7	361	84.3	2.43 (1.71, 3.46)	
Enrolment in community-based health insurance (CBHI)	Yes	81	7.1	1055	92.9	1	0.000⁣^∗^
	No	59	18.4	261	81.6	2.94 (2.05, 4.23)	
Means of transport to the health facility	On foot	112	9.6	1058	90.4	1.62 (0.80, 3.27)	0.176
	Animal	19	13.7	120	86.3	2.43 (1.06, 5.57)	0.036⁣^∗^
	Vehicle	9	6.1	138	93.9	1	
Waiting time at health facility (minutes)	≤ 30 min	35	5.1	658	94.9	1	0.000⁣^∗^
	> 30 min	105	13.8	658	86.2	3.00 (2.02, 4.46)	
Respondent knows health extension worker (HEW)	Yes	85	7.0	1132	93.0	1	0.000⁣^∗^
	No	55	23.0	184	77.0	3.98 (2.74, 5.78)	
Respondent's knowledge about immunization (mean = 5.70, SD = 2.62)	Poor	112	16.7	558	83.3	5.43 (3.54, 8.34)	0.000⁣^∗^
	Good	28	3.6	758	96.4	1	
Respondent's attitude towards immunization	Negative	45	18.3	201	81.7	12.54 (5.25, 29.91)	0.000⁣^∗^
	Neutral	89	10.3	779	89.7	6.40 (2.77, 14.77)	0.000
	Positive	6	1.8	336	98.2	1	
Respondent received health education at health facility	Yes	33	3.5	899	96.5	1	0.000⁣^∗^
	No/do not remember	107	20.4	417	79.6	6.99 (4.65, 10.5)	

⁣^∗^Candidates for multiple binary logistic regression.

**Table 5:** Factors independently associated with zero-dose vaccination in children aged 12–35 months, the West Arsi Zone, 23 June–17 August 2023.

**Variables**		**COR (95% CI)**	**AOR (95% CI)**	**p** ** value**
Availability of refrigerator at health post	Yes	1	1	0.003⁣^∗^
	No	1.41 (0.99, 2.01)	2.21 (1.30, 3.74)	
Presence of social mobilization for immunization	Yes	1	1	0.001⁣^∗^
	No/do not remember	5.25 (3.55, 7.77)	2.94 (1.59, 5.43)	
Sex of index child	Male	1	1	0.045⁣^∗^
	Female	1.55 (1.09, 2.20)	1.61 (1.01, 2.57)	
Age of respondent	15-24	3.77 (2.64, 5.39)	1.97 (1.08, 3.59)	0.027⁣^∗^
	25-34	1	1	
	35-44	1.35 (0.87, 2.11)	0.87 (0.48, 1.57)	0.638
	≥45	2.82 (1.43, 5.58)	1.13 (0.41, 3.14)	0.813
ANC during pregnancy of index child?	Yes	1	1	0.046⁣^∗^
	No	16.12 (10.69, 24.31)	2.45 (1.08, 6.88)	
Index child's place of birth	HF	1	1	0.000⁣^∗^
	Home/street	8.0 (5.15, 12.43)	3.95 (2.22, 7.03)	
Waiting time at health facility	≤ 30 min	1	1	0.005⁣^∗^
	> 30 min	3.00 (2.02, 4.46)	2.19 (1.27, 3.75)	
Respondent knows health extension worker (HEW)	Yes	1	1	0.043⁣^∗^
	No	3.98 (2.74, 5.78)	1.39 (1.07, 2.53)	
Respondent's knowledge about immunization	Poor	5.43 (3.54, 8.34)	3.22 (1.88, 5.52)	0.000⁣^∗^
	Good	1	1	
Respondent's attitude towards immunization	Negative	4.21 (2.67, 6.62)	5.92 (2.14, 16.41)	0.001⁣^∗^
	Neutral	2.85 (1.86, 4.36)	3.85 (1.51, 9.79)	0.005⁣^∗^
	Positive	1	1	1
Respondent received health education at health facility	Yes	1	1	0.014⁣^∗^
	No/do not remember	6.99 (4.65, 10.5)	2.00 (1.15, 3.50)	
Enrolment in community-based health insurance (CBHI)	Yes	1	1	0.018⁣^∗^
	No	2.94 (2.05, 4.23)	1.89 (1.11, 3.21)	
Wealth index (calculated from 21 asset variables)	Poorest	2.51 (1.32, 4.75)	3.80 (1.40, 10.33)	0.009⁣^∗^
	Middle lower	2.29 (1.17, 4.47)	2.69 (1.15, 7.62)	0.042⁣^∗^
	Middle	1.52 (0.76, 3.05)	1.65 (0.61, 4.52)	0.327
	Middle upper	3.34 (1.78, 6.26)	3.97 (1.60, 9.85)	0.003⁣^∗^
	Richest	1	1	

⁣^∗^Statistically significant at *p* < 0.05.

## Data Availability

The data that support the findings of this study are available from the corresponding author upon reasonable request.

## Nomenclature

ABMHSU: Andersen's behavioral model of health service use
ANC: antenatal care
CBHI: community-based health insurance
DHIS-2: District Health Information Software 2
EDHS: Ethiopian Demographic and Health Survey
EPI: expanded program on immunization
HDA: Health Development Army
HE: health education
HEWs: health extension workers
HF: health facility
HH: household
HI: health information
HTR: hard-to-reach
km: kilometer
ODK: Open Data Kit
PHF: public health facility
PSNP: Productive Safety Net Program
RCSs: rapid convenient surveys
TT: tetanus toxoid
WDA: Women Development Army
